# The efficacy of cognitive interventions on the performance of instrumental activities of daily living in individuals with mild cognitive impairment or mild dementia: protocol for a systematic review and meta-analysis

**DOI:** 10.1186/s13643-019-1135-0

**Published:** 2019-08-28

**Authors:** Nikki Tulliani, Michelle Bissett, Rosalind Bye, Katrina Chaudhary, Paul Fahey, Karen P. Y. Liu

**Affiliations:** 1 School of Science and Health, Western Sydney University, Penrith, NSW Australia; 2 Translation Health Research Institute, Western Sydney University, Penrith, NSW Australia; 3 School of Allied Health Sciences, Griffith University, Gold Coast, QLD Australia

**Keywords:** Cognitive interventions, Instrumental activities of daily living, Mild cognitive impairment, Mild dementia, Protocol for systematic review

## Abstract

**Background:**

Cognitive changes associated with mild cognitive impairment or mild dementia can lead to difficulties in completing instrumental activities of daily living. The ability to live independently at home and in the community is often compromised due to the inability to complete these activities. Cognitive interventions have been reported as beneficial in maintaining or improving cognitive functions among this group of adults. However, the effectiveness of different types of cognitive interventions on the performance of instrumental activities of daily living in older adults with mild cognitive impairment and mild dementia is not well established. The aim of this paper is to develop a protocol for a systematic review and meta-analysis to investigate the effectiveness of cognitive interventions in maintaining or improving the performance of instrumental activities of daily living in individuals with mild cognitive impairment or mild dementia.

**Methods:**

Randomised control studies which investigate the effectiveness of cognitive interventions on the performance in instrumental activities of daily living for older adults with mild cognitive impairment and mild dementia will be sought. A systematic search will be conducted in five databases: CINAHL, MEDLINE, EMBASE, PsycINFO and Cochrane Central Register of Controlled Trials. The search strategy was developed with assistance from a health science librarian. Two independent reviewers will perform the study selection and data extraction. Quality assessment will be implemented using the Physiotherapy Evidence Database (PEDro) scale. A narrative synthesis of the findings will be used to report outcomes of all included studies. If appropriate, a meta-analysis will combine the results of individual studies.

**Discussion:**

This systematic review and meta-analysis will determine the effectiveness of cognitive interventions in maintaining or improving the performance of IADL in individuals with MCI or mild dementia. It is anticipated that the results will inform rehabilitation professionals of the most effective cognitive interventions to be implemented into clinical practice. It will potentially provide substantial benefit to both the persons with MCI or dementia and the health care system by keeping more people out of full-time care and allowing those in full-time care to require less intensive support.

**Systematic review registration:**

PROSPERO CRD42016042364

## Background

Daily activities can be subcategorised as either basic or instrumental activities. Basic daily activities (BADL) are activities that are concerned with taking care of one’s own body and encompass 10 categories: bathing/showering, bowel and bladder management, dressing, eating, feeding, functional mobility, personal device care, personal hygiene and grooming, sexual activity and toilet hygiene [1]. Instrumental activities of daily living are more complex daily activities. They encompass 12 categories: care of others, care of pets, child-rearing, communication management, driving and community mobility, financial management, health management and maintenance, home establishment and management, meal preparation and clean-up, religious observance, safety and emergency maintenance, and shopping [1]. Engagement in these activities is required for people to participate in home and community life.

The ability to complete BADL usually remains intact among those with MCI or mild dementia [2, 3]. Impairment in BADL activities of daily has been shown to manifest after impairments of instrumental activities of daily living (IADL) [4]. There is a positive relationship between cognitive function and performance of IADL [5–10]. IADL demand more complex neuropsychological processing than BADL and are more susceptible to the subtle deterioration associated with cognitive decline [11]. It has been demonstrated that deterioration in memory and other cognitive functions may result in problems performing IADL, and thus, impact on people’s ability to independently live in the community [12–14]. For example, de Paula et al. [6] examined the subdomains of memory and executive functioning, finding that episodic memory and executive function correlated with IADL performance in domestic chores, telephone use, meal preparation and laundry (ps < .p5) as well as significantly predicted performance in financial management, shopping, medication management and using transportation (ps < .05). Similar results were found in Cahn-Weiner et al. [15] that memory and executive function were significantly associated with IADL performance (*p* = .001). Similarly, Farias et al. [16] also revealed that change in executive function was associated with a change in IADL performance (*p* < .001). Thus, a greater degree of decline in memory and executive function were associated with greater functional decline. A systematic review [17] examining IADL performance of individuals with MCI compared with cognitively healthy individuals and people with dementia found that people with MCI had intermediate functional performance in more complex tasks requiring higher cognitive demand such as telephone use, medication management and keeping appointments between cognitively healthy controls people with mild dementia, particularly.

As part of the normal ageing process, older adults may experience deterioration in cognition in which their cognitive functioning negatively affects their ability to perform IADL [18]. These cognitive changes appear to be greater in individuals with MCI and even more significant in individuals with dementia [17, 19, 20]. MCI and dementia are classified as neurocognitive disorders, whereby there is evidence of an acquired cognitive decline in one or more neurocognitive domains [21]. Deficits may be present in any of the six cognitive domains outlined in the Diagnostic and Statistical Manual of Mental Disorders (DSM-5). These domains are complex attention, executive function, learning and memory, language, perceptual-motor function and social cognition. Cognitive interventions, which address the various cognitive domains, may be vital to maintaining or preventing a decline of cognitive function and subsequent IADL performance in individuals in the pre-clinical or early stages of dementia.

Cognitive interventions include (1) cognitive training, (2) cognitive rehabilitation and (3) cognitive stimulation [22]. These three intervention approaches feature in current literature relating to individuals with MCI and dementia as interventions targeting specific cognitive functions or interventions targeting functional performance in activities including IADL [22, 23].

Cognitive *training* consists of practising cognitive tasks, focussing on improving cognitive functions in areas such as memory, attention, problem solving and calculation. Cognitive training aims to improve cognitive functions in one or more cognitive domains [22, 24].

Unlike cognitive *training*, cognitive *rehabilitation* does not aim to improve cognitive functions specifically. Instead, the aim of cognitive rehabilitation is to address the problems in daily activity performance as a result of the decline in cognitive functions. Cognitive rehabilitation focuses on identifying goals to enhance daily activity performance, providing a tailored intervention for each person. Interventions often include providing compensatory and adaptive strategies and are targeted at improving performance in specific daily activities. Examples of cognitive rehabilitation include memory retrieval techniques, activity or environment modification and errorless learning [22, 25].

Cognitive *stimulation* is another intervention strategy that promotes engagement in activities to stimulate general cognitive and social functioning in a non-specific manner. Examples of cognitive stimulation include participating in group discussions, book clubs, quizzes and trivia, or music-related activities [24, 25].

A therapy program that utilises a combination of cognitive training, rehabilitation and/or stimulation with individuals with MCI or early stages of dementia may have the greatest benefit because they continue to retain the ability and the cognitive capacity associated with learning and applying new skills [26]. These three cognitive interventions are commonly adopted to assist people living with MCI or dementia as there is currently no proven curative therapy [23, 27]. Therefore, early cognitive intervention becomes vital in maintaining cognitive functions and daily activity performance in older adults.

Due to varying criteria for MCI and the difficulties with diagnosis, it is difficult to determine the prevalence of the disease [28–30]. A Canadian Health and Aging study reported an estimated population prevalence of 1.03 to 3.02% when adopting different definitions for MCI [29]. The range in prevalence between 3% and 19% was also demonstrated in a population-based epidemiological study [31]. Another review [32] reported prevalence estimates for MCI ranging from 16 to 20%.

Although the exact prevalence of MCI is difficult to determine, it has become widely recognised that the mild cognitive changes associated with MCI are a probable transitional period between normal ageing and a clinical diagnosis of dementia [33–36]. A study of 133 individuals with MCI and found a conversion rate from MCI to Alzheimer’s disease type dementia to be a staggering 30.5% [37]. According to the World Health Organization, around 50 million people are living with dementia. The total number of people with dementia is expected to increase to 82 million in 2030 and 152 million by 2050 [38]. In 2015, it was estimated that an equivalent value of 1.1% of global gross domestic product would be required to cover the total global societal cost of dementia which was estimated to be USD 818 billion [38]. Mild cognitive impairment, therefore, can constitute a substantial economic burden for public health systems.

With the increasing prevalence of cognitive impairment and dementia in the aging population [39–42], there is a need to review the efficacy of cognitive interventions to maintain or improve function. To date, reviews have reported the effectiveness of cognitive interventions on cognitive function, particularly on memory. Although individuals with MCI may have preserved functional abilities, it is well established that they may experience subtler difficulties such as making more errors or needing more time than healthy individuals to perform complex IADL. There has not been a systematic review examining the effectiveness of cognitive interventions directly on IADL across the continuum of cognitive decline from MCI to mild dementia. For example, systematic reviews by Simon et al. [25] and Jean et al. [43], both examined the impact of cognitive interventions for MCI on daily activity performance but only as a secondary outcome, and the results on specific IADL were not reported.

This current review will extend previous systematic reviews by including the effectiveness of cognitive interventions on the performance of IADL in all subtypes of MCI and mild dementia. The associated decline in cognition and decreased performance of IADL are not only associated with reduced independence and quality of life [7], but also have far-reaching economic implications and increased health care costs associated that may present challenges to the current health care system [7, 44]. For example, the total direct cost of dementia was $8799 million in Australia and is projected to increase two-fold in 20 years’ time [45]. Most of the direct cost goes to hospitalisation, attending general practitioners and specialists and providing care. Facilitating performance of IADL in people with MCI or mild dementia is an important strategy to aid successful community-based living and reduce the need for additional care and health services. This systematic review and meta-analysis will summarise the available evidence regarding the efficacy of cognitive interventions on the performance of IADL in adults with MCI or mild dementia and highlight areas for further research into cognitive interventions to promote IADL functioning for independent living.

## Objectives

The objectives of this review are to identify, evaluate and synthesise all available randomised controlled trials of cognitive interventions (cognitive training, cognitive rehabilitation and cognitive stimulation) targeted at maintaining or improving IADL performance in older adults with MCI or mild dementia.

## Methods

A systematic review utilising narrative synthesis and a meta-analytical approach will be conducted according to the Preferred Reporting Items for Systematic Reviews and Meta-analysis (PRISMA) guidelines [46]. The protocol of this systematic review has been registered in PROSPERO (registration number CRD42016042364).

### Eligibility criteria

#### Types of studies

Studies included will report (1) cognitive interventions used with older adults with MCI or mild dementia and (2) performance in at least one IADL at baseline and post-treatment. Studies that are published in the English language in a peer-reviewed journal will be eligible. All randomised controlled trials with participants entering into both arms of the trial will be reported. Comparative studies with and without concurrent controls, such as non-randomised experimental trials, cohort studies, case-control studies, single-arm studies and case series, will be excluded from this review.

#### Types of participants

Studies with participants aged 60 or above, residing in either the community or within a residential aged care setting, and with a diagnosis of MCI or mild dementia as outlined by one of the following criteria, will be included:

- World Health Organization’s International Classification of Diseases code [47];
- National Institute of Neurological and Communicative Disorders and Stroke and the Alzheimer’s Disease and Related Disorders Association criteria [48];
- American Psychiatric Association’s Diagnostic and Statistical Manual of Mental Disorders [21];
- Clinical Dementia Rating scale [49];
- Blessed Dementia Rating Scale [50];
- Petersen’s Diagnostic Criteria for MCI [51];
- Mayo Clinic Diagnostic Criteria for MCI [35];
- National Institute on Aging and the Alzheimer’s Association [52]; or
- International Working Group on MCI Diagnostic Criteria [53].

Studies with the study sample under 60 years of age or with moderate to severe dementia will be excluded. Studies reporting participants with a diagnosis of MCI or mild dementia utilising an alternative diagnostic criterion will be considered if the diagnostic criteria are standardised, valid and reliable. Two reviewers (NT and KL) will review the available literature to reach a consensus as to whether studies utilising other diagnostic criteria will be included.

#### Types of interventions and comparisons

Cognitive interventions of interest are cognitive training, cognitive rehabilitation and cognitive stimulation. Delivery of these interventions is not limited to a specific mode. They can include face-to-face, computer-administered, individual or group interventions. Interventions delivered in any setting, inclusive of inpatient and outpatient hospital settings, community-based programs, rehabilitation settings, adult day support facilities and residential aged care facilities, will be included.

Interventions may be compared with active controls (for example, another rehabilitation intervention, such as an exercise program [54], caregiver training [55] or dietary changes [56]) or an inactive control group (for example, wait-list control or standard care). Studies which compare two cognitive interventions without control or standard care arm will be excluded. Interventions of any duration, frequency, intensity and delivery will be included.

#### Types of outcome measures

The outcome of interest is improved or maintained IADL performance. Studies must include at least one outcome measure assessing the performance of one or more IADL. The IADL will be included if it falls under one of the following 12 categories: care of others, care of pets, child-rearing, communication management, driving and community mobility, financial management, health management and maintenance, home establishment and management, meal preparation and clean-up, religious and spiritual activities and expression, safety and emergency maintenance, and shopping [1]. Outcomes can be performance-based assessed by therapists, self-reported by the participant or informant reported by a caregiver or significant other. Both standardised and non-standardised assessments will be included.

### Information sources

Search strategy and study selection searches will be undertaken in OVID SP versions of MEDLINE and EMBASE, EBSCO versions of CINAHL and PsycINFO and the Cochrane Central Register of Controlled Trials.

In addition to the studies retrieved from the above databases, reference lists of all included studies and other reviews on the topic will be searched to identify any additional potentially relevant studies for inclusion. The Cochrane database and the Journal of Ageing Research Reviews will also be searched to identify reviews in similar areas.

### Search strategy

Search terms are based on terms from existing reviews, for example, Bahar-Fuchs et al. [22], Kelly et al. [24], Simon et al. [25], Li et al. [57]. The MEDLINE (Ovid) search strategy is detailed in the Appendix section. This search strategy will be tailored to the thesaurus or controlled vocabulary and search syntax of each database. Publications will be limited to human studies, published in English peer-reviewed journals, between March 2009 and March 2019. Results from the database searches will be exported and managed in an Endnote reference management software. The searches will be re-run just before final analyses and further studies retrieved for inclusion if found.

Study selection and data extraction process

**Fig. 1:**
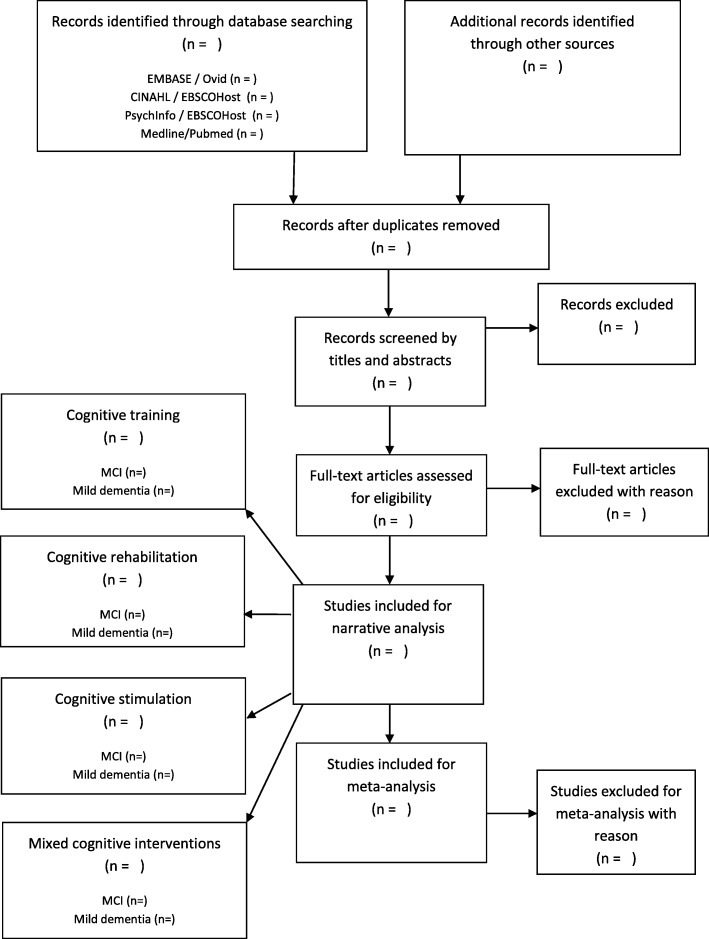
Flow diagram of the study selection process based on the PRISMA guidelines

Two independent reviewers (NT and KL) will be involved in the study selection and data extraction. The study selection process will be in accordance with the PRISMA guidelines (Fig. 1). A data extraction form will be developed and piloted independently by two reviewers (NT and KL) on 10% of the identified studies prior to use and modified as required.

During initial screening, all papers with study titles and abstracts viewed as potentially eligible by at least one of the two reviewers will be retained for full review. A full review will be conducted by the two reviewers independently. Reasons for inclusion and exclusion will be recorded. Once eligible papers have been identified, data will be extracted independently by the two reviewers. Disagreements relating to eligibility and differences in data extraction will be resolved by discussion between the two reviewers to reach a consensus. A third reviewer (MB) will resolve any difference identified between the two reviewers.

When information is not available within the selected studies, contacting the authors via email will be attempted. If the information is not available after this process, the most conservative estimates will be made using available data, i.e., at the lower 95% confidence interval.

### Methodological critique of evaluation research

The methodological quality of the included studies will be assessed using the Physiotherapy Evidence Database (PEDro) scale [46]. The risk of bias assessment will be critiqued independently by two reviewers (NT and KL); a third reviewer (MB) will resolve any differences in opinion.

### Data extraction

The following data will be extracted:

1. Participant information: sample size, mean age, diagnosis and diagnostic criteria utilised and baseline cognitive score if indicated.
2. Methods of each study: study design, treatment setting and methodological limitations reported.
3. Type of interventions: aim of intervention, type of cognitive intervention, duration of treatment (duration of sessions, frequency of sessions, period of intervention, total hours of intervention), method of intervention delivery and description of control group intervention.
4. Outcome measures: performance of IADL at pre- and post-intervention period and post-intervention follow-up if data is available.
5. Results of the studies: impact (if any) in the performance of IADL for experimental and control groups.

If identified studies include mixed cohorts (including healthy adults, MCI or dementia, or combining with people younger than 60), an attempt will be made to contact the corresponding author to request results for just the eligible participants. If not, these studies will be excluded from the review.

### Synthesis and analysis of results

A narrative synthesis of the findings will be used to report outcomes of all included studies. This synthesis will be formatted around study type, sample size, participant characteristics, outcomes and outcome measures. The context of intervention on type, quantity, frequency and/or duration of therapy will be described. ‘Summary of findings’ tables will be created to provide key information regarding evidence quality, a summary of available data on outcomes and the degree of the effectiveness of interventions. The synthesis of findings will be in accordance with the Centre for Reviews Dissemination [58]. The Economic and Social Research Council guidance report [59, 60] will be used as a framework for a narrative synthesis. This framework consists of four key elements: (1) the development of a theory of how the intervention works, why and for whom, (2) the development of a preliminary synthesis of findings of included studies, (3) the identification of relationships within and between studies and (4) the assessment of the strength of the synthesis.

### Measures of treatment effectiveness

Each study and outcome measure will be assessed for suitability for meta-analysis. Two reviewers (NT and KL) will evaluate and identify the major outcome measure that represents the main outcome of each study for meta-analysis. The treatment effects, based on pooled data from individual studies, will be recorded. Means and standard deviations (SDs) or medians at pre-, post-intervention and follow-up assessments will be extracted from each study. If the means and SDs or medians are not available, the corresponding author will be contacted for the available data. If further information is not available, medians will be used to replace means, and baseline SD will be used as an estimate of SD at follow-up. If the required data cannot be retrieved, the study will be excluded from the meta-analysis.

A separate analysis will be performed on studies using cognitive training, cognitive rehabilitation and cognitive stimulation. Depending on available data extracted from each study, identical and non-identical outcome measures will be combined for analysis with the standardised mean differences (SMD). In addition, the *p* value and 95% confidence intervals (CI) will be reported. If both categorical and numeric measures are used in the studies, two separate meta-analyses will be conducted. Heterogeneity will be assessed using the *I*^2^ statistic. Meta-analysis with an *I*^2^ > 40% will be considered to have substantial heterogeneity, and appropriate warnings will be given against overinterpretation of these results [61]. The sample size will be weighted downwards by the estimated design effect. The design effect will be estimated using the intra-class correlation coefficient (ICC) if provided. If the ICC is not provided, it will be estimated from the wider literature. The effect size of individual outcome measures will be calculated using Hedge’s *g*, with included adjustments for small sample size. The analysis will be performed using the ‘metafor’ package in R software, where the random effect model with 95% CI will be used [62].

The robustness and generalisability of the results will be explored by a variety of sensitivity analyses such as excluding the lower quality studies and studies from less developed or developing countries. An experienced statistician will assist with the completion of the meta-analysis.

### Confidence in cumulative evidence

The quality of evidence for primary outcomes from each of the studies included in the review will be assessed using the Grades of Recommendation, Assessment, Development, and Evaluation (GRADE) approach [63] by the first author (NT). A second author (KL) will verify the ratings; any disagreements will be discussed and reconciled with a third author (MB). The meta-analysis will be assessed in relation to the quality of the evidence scored in the five domains specified within GRADE: limitations in study design and/or execution (risk of bias), inconsistency of results, indirectness of evidence, imprecision of results and publication bias [63]. The quality of each study will be rated as high, moderate, low or very low according to the level of confidence in where the effect lies in relation to the estimated effect.

### Ethics and dissemination

Ethical approval is not required for this study, as this systematic review did not directly or indirectly involve human participants. Data will be extracted from publicly available published literature, and the analysis is secondary to this. Findings from this systematic review will be submitted as a manuscript for peer review in an appropriate journal. Findings will also be presented to clinicians and researchers at relevant conferences.

### Amendments

If amendments to the protocol outlined are required, the date of the amendment, the change required and rationale for the change will be documented in a protocol addendum and in the final report of the systematic review.

## Discussion

Dementia is an overarching term used to describe a group of diseases associated with an observable decline in cognitive functioning, and individuals living with these conditions often have difficulty with daily activities that affect their independent living. Due to the multiple clinical presentations and underlying etiologies associated with dementia and MCI, there is currently no curative treatment available for the underlying diseases that cause these syndromes [64]. This systematic review and meta-analysis will determine the effectiveness of cognitive interventions in maintaining or improving the performance of IADL in individuals with MCI or mild dementia. It is anticipated that the dissemination of results will inform rehabilitation professionals on the most effective cognitive interventions for clinical practice. It will potentially provide substantial benefit to both people living with MCI or dementia and the health care system by keeping more people living in the community rather than full-time residential care and allowing those in full-time residential care to require less intensive support. Moreover, it can inform and encourage the development of policies and guidelines for the support of these individuals. Results and policy recommendations will be presented at rehabilitation conferences and policy forums. In addition, our description of all recent research in this topic area will identify if and where further research is required.

## Data Availability

The datasets used and/or analysed during the current study are available from the corresponding author on reasonable request.

## Appendix

Database: Ovid MEDLINE(R) Epub Ahead of Print, In-Process & Other Non-Indexed Citations and Ovid MEDLINE(R) without Revisions <1996 to February 09, 2018> Search Strategy:

-----------------------------------------------------------------

1. exp Dementia/ or exp Cognitive Dysfunction/ or exp Alzheimer Disease/or exp Cognition Disorders/ (157088)
2. ((mild adj dementia) or MCI or dementia).tw. (81709)
3. ((cognitive adj2 dementia) or CIND).tw. (1944)
4. memory disorders/ or exp amnesia/ (19643)
5. ((age-associated adj2 impairment) or AAMI).tw. (554)
6. ((age-related adj2 impairment) or (memory adj impairment) or (memory adj decline) or (memory adj loss) or (impaired adj memory)).tw. (13051)
7. (alzheimers or (cognitive adj decline) or (memory adj decline)).tw. (102555)
8. 1 or 2 or 3 or 4 or 5 or 6 or 7 (238990)
9. exp Cognitive Therapy/ or (cognitive adj therap$).tw. (22154)
10. (cognitive and (intervention or training or techniques or restoration or retraining or re-training or stimulation or rehabilitation or remediation)).tw. (48052)
11. exp Neurological Rehabilitation/ or Rehabilitation/ (15310)
12. exp Mental Recall/ or (mental adj stimulation).tw. (20456)
13. (task adj2 training).tw. (1275)
14. exp Occupational Therapy/ or (occupational adj rehabilitation).tw. (6356)
15. ((sensory adj stimulation) or (reminiscence adj therapy)).tw. (1854)
16. exp "Imagery (Psychotherapy)"/ or (mental adj imagery).tw. (2438)
17. (skill and (acquisition or retention)).tw. (3582)
18. exp Learning/ or (memory adj training).tw. (226476)
19. (memory and (encoding or retrieval)).tw. (15907)
20. ((task adj2 training) or (functional adj task)).tw. (1586)
21. ((guided adj imagery) or (motor adj imagery)).tw. (2513)
22. Visual Perception/ or Cues/ (57601)
23. (visualisation or cues).tw. (55718)
24. 9 or 10 or 11 or 12 or 13 or 14 or 15 or 16 or 17 or 18 or 19 or 20 or 21 or 22 or 23 (372351)
25. exp "Activities of Daily Living"/ or ADL.tw. (51146)
26. ((Instrumental adj3 living) or (activities adj living) or IADL).tw. (2617)
27. (functional and (performance or ability or status)).tw. (136152)
28. (daily and (task or activities)).tw. (47564)
29. ((complex adj activities) or (task adj performance) or (day adj2 activities)).tw. (9592)
30. 25 or 26 or 27 or 28 or 29 (216937)
31. 8 and 24 and 30 (4691)
32. limit 31 to (english language and yr="2008 -Current") (3129)
33. limit 32 to randomized controlled trial (370)

## Abbreviations

CI: Confidence intervals
IADL: Instrumental Activities of Daily Living
MCI: Mild cognitive impairment
*P* value: Probability value
PEDro: Physiotherapy Evidence Database
PRISMA: Preferred Reporting Items for Systematic Reviews and Meta-Analysis
SD: Standard deviations
SMD: Standardised mean differences
